# Mortuary and Thermometrical Reports

**Published:** 1880-09

**Authors:** 


MORTUARY AND THERMOMETRICAL REPORTS.—It will be seen that these reports were omitted in the previous issue, and that those for July appear in this, the September number of the Journal.

The omission was caused by the issue going to press ere the "report," for July could be compiled in the Health office. They will henceforth be published in the order observed in the present number, and for all practical and useful purposes, will be just as acceptable, when the volume is completed and indexed, as any other, that might be adopted. That these "reports" will be found valuable in the future, far beyond the confines of the city in which we live there cannot be a shadow of doubt; and in a few years, will alone, much exceed in value the present subscription price of the Journal, to all educated physicians and sanitarians. *Both* reports are *official and reliable.*

